# Cardiovascular outcomes associated with treatment of type 2 diabetes in patients with ischaemic heart failure

**DOI:** 10.1002/ehf2.13910

**Published:** 2022-03-23

**Authors:** Thomas R. Godec, Daniel I. Bromage, Mar Pujades‐Rodriguez, Antonio Cannatà, Arturo Gonzalez‐Izquierdo, Spiros Denaxas, Harry Hemingway, Ajay M. Shah, Derek M. Yellon, Theresa A. McDonagh

**Affiliations:** ^1^ Department of Medical Statistics, Faculty of Epidemiology and Population Health The London School of Hygiene & Tropical Medicine London UK; ^2^ School of Cardiovascular Medicine and Sciences King's College London British Heart Foundation Centre of Excellence, James Black Centre 125 Coldharbour Lane London SE5 9NU UK; ^3^ Leeds Institute of Health Sciences University of Leeds Leeds UK; ^4^ Institute of Health Informatics University College London London UK; ^5^ Health Data Research UK London University College London London UK; ^6^ The National Institute for Health Research University College London Hospitals Biomedical Research Centre University College London London UK; ^7^ The Hatter Cardiovascular Institute University College London London UK

**Keywords:** Heart failure, Ischaemic cardiomyopathy, Metformin, Outcomes, Type 2 diabetes, Antidiabetic agents

## Abstract

**Aim:**

The optimal strategy for diabetes control in patients with heart failure (HF) following myocardial infarction (MI) remains unknown. Metformin, a guideline‐recommended therapy for patients with chronic HF and type 2 diabetes mellitus (T2DM), is associated with reduced mortality and HF hospitalizations. However, worse outcomes have been reported when used at the time of MI. We compared outcomes of patients with T2DM and HF of ischaemic aetiology according to antidiabetic treatment.

**Methods and results:**

This study used linked data from primary care, hospital admissions, and death registries for 4.7 million inhabitants in England, as part of the CALIBER resource. The primary endpoint was a composite of cardiovascular mortality and HF hospitalization. The secondary endpoints were the individual components of the primary endpoint and all‐cause mortality. To evaluate the effect of temporal changes in diabetes treatment, antidiabetic medication was included as time‐dependent covariates in survival analyses. The study included 1172 patients with T2DM and prior MI and incident HF between 3 January 1998 and 26 February 2010. Five hundred and ninety‐six patients had the primary outcome over median follow‐up of 2.53 (IQR: 0.98–4.92) years. Adjusted analyses showed a reduced hazard of the composite endpoint for exposure to all antidiabetic medication with hazard ratios (HRs) of 0.50 [95% confidence interval (CI): 0.42–0.59], 0.66 (95% CI: 0.55–0.80), and 0.53 (95% CI: 0.43–0.65), respectively. A similar effect was seen for all‐cause mortality [HRs of 0.43 (95% CI: 0.35–0.52), 0.57 (95% CI: 0.46–0.70), and 0.34 (95% CI: 0.27–0.43), respectively].

**Conclusions:**

When considering changes in antidiabetic treatment over time, all drug classes were associated with reduced risk of cardiovascular mortality and HF hospitalization.

## Introduction

Type 2 diabetes mellitus (T2DM) and heart failure (HF) frequently coexist. Metformin remains a guideline‐recommended therapy for the treatment of T2DM in HF in European guidelines.^1^ Numerous observational studies have reported beneficial outcomes of metformin in HF, including reductions in all‐cause mortality and HF hospitalization.^2^ However, findings from studies investigating metformin use at the time of myocardial infarction (MI) are inconclusive, with some reporting worse outcomes.^3^, ^4^, ^5^, ^6^ Similar concerns have been raised for other antidiabetic agents, including sulfonylureas and insulin. Despite this, no studies have specifically investigated the relationship between antidiabetic treatment and outcomes in patients with T2DM and HF following MI. Moreover, existing evidence is limited by lack of control for changes in medication use over time. Using national linked electronic health records from primary care and hospitalizations, which include prescribed medication in primary care, we investigated whether antidiabetic drug administration and changes over time were associated with cardiovascular outcomes and all‐cause mortality in patients with T2DM and HF of ischaemic aetiology.

## Methods

### Study design and data sources

This study used linked longitudinal electronic health records from the Clinical Practice Research Datalink (CPRD) and Hospital Episode Statistics (HES) and cause‐specific mortality from the Office for National Statistics (ONS) in England, accessed through the CALIBER programme (https://www.ucl.ac.uk/health‐informatics/caliber).^7^ Further methodological details are provided in the supporting information. This was a prospective cohort study, and *Table* *S1* summarizes the STROBE and RECORD checklists for reporting on observational research.^8^, ^9^

### Study population and exposure definition

The study included all patients with diagnosis of HF, T2DM, and non‐fatal MI (either STEMI or non‐STEMI) recorded before or on the same day as HF, between January 1998 and October 2010. Diagnoses of HF, T2DM, and MI were identified in CPRD and HES using previously described and validated phenotyping algorithms.^10^, ^11^ Eligibility criteria for study inclusion were no history of HF prior to the study start date, at least one prescription of an oral antidiabetic medication following the index HF diagnosis and before the event of interest, a minimum of 1 year of follow‐up since practice registration and since the date on which the data from their CPRD practice were deemed to be of acceptable quality, and to be 18 years of age or above at the time of HF diagnosis. During follow‐up, for each drug class, continuous exposure was defined if repeat prescriptions were issued within 90 days and periods of non‐exposure started after 90 days. Patients managed by diet alone were excluded, and patients taking thiazolidinediones were also excluded due to established safety issues.^12^, ^13^ Patients were permitted to be on more than one antidiabetic medication sequentially or concurrently.

### Baseline characteristics

As a surrogate for glycaemic control at baseline, the level of glycosylated haemoglobin (HbA1c) was determined when this was recorded within a year prior to index HF. For each patient, data on baseline comorbidities and cardiovascular risk factors, including age, sex, body mass index, index of multiple deprivation score, smoking status, systolic and diastolic blood pressure, cholesterol, and history of cardiovascular disease, were defined in previous CALIBER research studies and identified in CPRD, HES, and ONS.^6^, ^14^ Further details are provided in the supporting information.

### Study endpoints

The primary endpoint was a composite of cardiovascular death and HF hospitalizations. Secondary endpoints were the individual components of the primary endpoint (cardiovascular death and HF hospitalizations) and all‐cause mortality.

### Statistical analyses

Demographic and baseline characteristics of the study population were calculated using means (SDs) for continuous variables or medians (IQRs) as appropriate, and counts (with percentages) for categorical variables. To evaluate the effect of temporal changes in diabetes treatment, antidiabetic medications were included as time‐dependent covariates in survival analyses, based on repeat prescription data as above. Subjects were censored at the time of death, leaving their GP practice or last date of data collection. Multivariable Cox proportional hazard regression was used to estimate hazard ratios for the effects of antidiabetic agents. Models were adjusted *a priori* for other antidiabetic drugs (included as time‐dependent variables) and established cardiovascular risk factors at baseline: sex, body mass index, and smoking status (all categorical variables), and baseline HbA1c, age, systolic blood pressure, and total serum cholesterol (continuous variables). Additionally, final models were adjusted for baseline covariates where evidence for their relationship with the outcome was found, as shown by a *P* < 0.2 in univariable analyses, or whose inclusion in the model resulted in a change in estimated effect of any of the four medication groups of >10%, while ensuring that there were at least 10 events per estimated model coefficient.

### Sensitivity analyses

Sensitivity analyses included, first, using a propensity score approach, as previously described.^6^ Briefly, a propensity score in relation to metformin use was predicted for each subject using baseline covariates that showed evidence of an association with the primary composite outcome in a logistic regression model.^15^, ^16^ The estimated propensity scores were then used to create inverse probability weights for each subject.^17^ These weights were used in a Cox proportional hazards regression model to estimate treatment group effects. Second, analysis of HF survivors at 30 days post‐index MI (i.e. those who left the GP practice, were ‘lost to follow‐up’ or had an event within 30 days were excluded), to distinguish between acute and chronic effects of antidiabetic medication and minimize the potential confounding effect of the previously observed association between metformin use at the time of MI and increased hazard of major adverse cardiovascular events.^6^ Further details are given in the supporting information. Third, we repeated the analysis using a 30 day window between prescriptions instead of 90 days to define continuous treatment. Fourth, HbA1c was included as a time‐dependent variable to examine the effect of diabetes control over time. Finally, we conducted a complete‐case analysis including only patients with complete covariate data to assess the effect of missingness.

## Results

### Antidiabetic agent use and patient baseline characteristics

**Figure 1:**
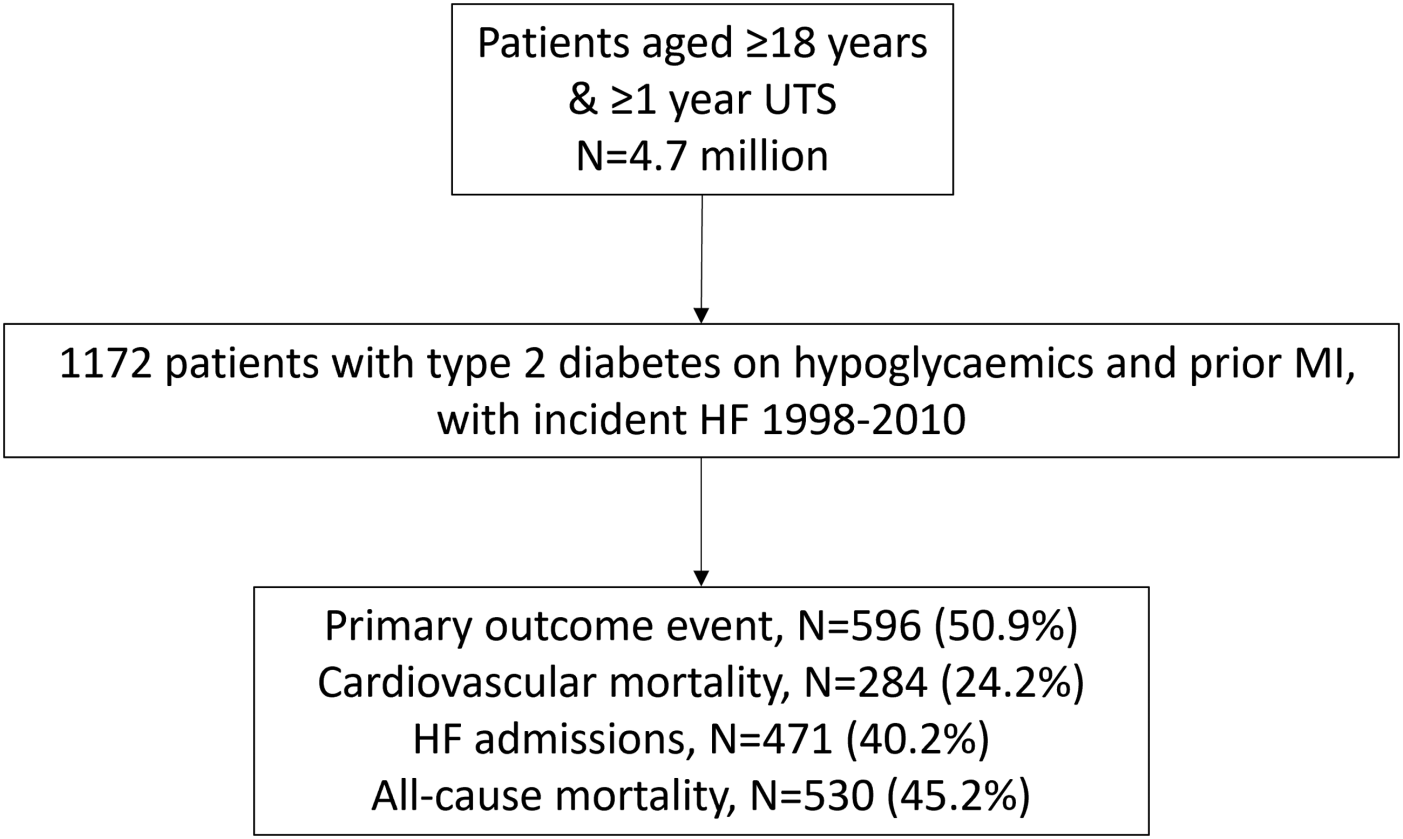
Study flow diagram. HF, heart failure; MI, myocardial infarction; UTS, up to standard.

**Figure 2:**
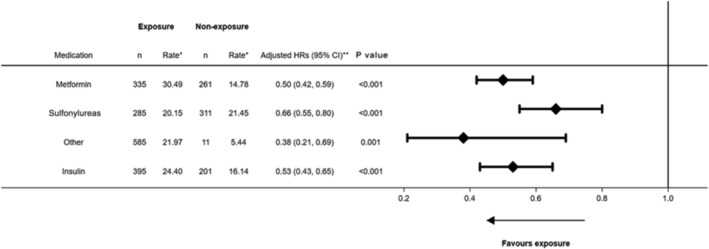
Number of events, event rates, and adjusted HRs (95% CI) with forest plot for time‐dependent periods of antidiabetic medication class exposure during follow‐up and the composite of cardiovascular death and HF hospitalization [primary endpoint]. *Per 100 person years; **Adjusted for each medication listed in the figure and the following baseline (time of index HF event) characteristics: age, sex, IMD category, BMI, smoking status, HbA1c, SBP, total cholesterol, HbA1c, history of CHD, history of ischaemic stroke, history of TIA, history of AAA, and history of PAD. Note: adjusted analysis performed on multiply imputed data. CI, confidence interval; HR, hazard ratio.

Out of 4.7 million patients in the linked CPRD‐HES‐ONS dataset, we identified 1172 eligible patients with T2DM on antidiabetic agents and with prior MI, who had incident HF during the study period (*Figure* *1*). Of these eligible patients, 854 (72.9%) took metformin, 790 (67.4%) a sulfonylurea, 81 (6.9%) ‘other’, and 550 (46.9%) insulin at some point during follow‐up. The median follow‐up time was 2.53 (IQR: 0.98–4.92) years, and total person years of follow‐up was 3731.14 (total exposure duration for each medication is given in *Table* *1*). A total of 596 (50.9%) had a primary outcome event during follow‐up (*Figure* *2*).

**Table 1:** Median (IQR) and total exposure time antidiabetic medication class during follow‐up

	Number of patients with some level of exposure during follow‐up (%), *N* = 1172	Median exposure time (IQR) in those with some level of exposure, years	Total exposure time (percentage of total follow‐up time), years
Metformin	854 (72.9)	1.81 (0.55–4.00)	2181.47 (58.5%)
Sulfonylureas	790 (67.4)	1.60 (0.50–3.52)	1865.76 (50.0%)
Other	81 (6.9)	2.32 (1.12–4.99)	261.70 (7.0%)
Insulin	550 (46.9)	2.38 (0.81–4.90)	1701.58 (45.6%)

IQR, interquartile range.

Total follow‐up time is 3731.14 years.

Patient baseline characteristics are shown in *Table* *2*. Patients' mean age was 71, and 37.1% were women. Median baseline HbA1c was 56 (IQR 49–69) mmol/mol. Approximately 74.5% and 71.0% of patients had been previously treated with metformin and sulfonylureas, respectively, while only 27.9% had previously received insulin.

**Table 2:** Baseline patient characteristics by primary outcome status, in patients with at least one prescription for an antidiabetic mediation during follow‐up

	Distribution of baseline characteristics (*N* = 1172)
Age (years), mean (SD)	71.26 (11.17)
Female, *n* (%)	435 (37.1)
IMD, *n* (%)	
<8.5 (least deprived)	187 (16.3)
8.5 to <34.18	740 (64.4)
≥34.18 (most deprived)	222 (19.3)
BMI (kg/m^2^), *n* (%)	
<20	26 (2.3)
20–25	217 (19.2)
25–30	427 (37.8)
30–35	290 (25.7)
≥35	169 (15.0)
Smoking status, *n* (%)	
Never‐smoker	498 (43.3)
Ex‐smoker	499 (43.4)
Current smoker	152 (13.2)
Systolic BP (mmHg), mean (SD)	137 (23)
Diastolic BP (mmHg), mean (SD)	75 (11)
HbA1c (mmol/mol), median (IQR)	56 (49–69)
Prior diabetes medication, *n* (%)	
Metformin, *n* (%)	873 (74.5)
Sulfonylureas, *n* (%)	832 (71.0)
Thiazolidinediones, *n* (%)	157 (13.4)
Acarbose, *n* (%)	72 (6.1)
DPP4 inhibitors, *n* (%)	10 (0.9)
GLP1 agonists, *n* (%)	2 (0.2)
Meglitinides, *n* (%)	2 (0.2)
Insulin, *n* (%)	327 (27.9)
HDL serum cholesterol (mmol/L), mean (SD)	1.17 (0.38)
Total serum cholesterol (mmol/L), mean (SD)	4.25 (1.25)
History of cardiovascular disease	
CHD, *n* (%)	666 (56.8)
Ischaemic stroke, *n* (%)	44 (3.8)
TIA, *n* (%)	81 (6.9)
PAD, *n* (%)	237 (20.2)
AAA, *n* (%)	219 (18.7)
Angina, *n* (%)	602 (51.4)

AAA, abdominal aortic aneurysm; BMI, body mass index; CHD, coronary heart disease; DPP, dipeptidyl peptidase; GLP, glucagon‐like peptide; HbA1c, haemoglobin A1c; HDL, high density lipoprotein; IMD, index of multiple deprivation; MI, myocardial infarction; NOS, not otherwise specified; PAD, peripheral arterial disease; TIA, transient ischaemic attack.

Patients with missing data were as follows: 43 for BMI, 23 for smoking status, 4 for IMD score, 7 for systolic and diastolic blood pressure, 300 for HbA1c, 191 for HDL cholesterol, and 47 for total cholesterol. *P* value is either from a *χ* ^2^ test if categorical or *t*‐test with unequal variances if continuous.

### Outcomes

Adjusted models showed a reduction in the hazard of the composite endpoint for periods on metformin, sulfonylureas, or insulin, with HRs of 0.50 (95% CI: 0.42–0.59), 0.66 (95% CI: 0.55–0.80), and 0.53 (95% CI: 0.43–0.65), respectively (*P* < 0.001 for all; *Figure* *2*) compared with periods of non‐use. This association was also found for the secondary outcomes of cardiovascular mortality and HF hospitalization (*Table* *3*). Similarly, diabetic drug treatment was associated with a reduction of all‐cause mortality, with HRs of 0.43 (95% CI: 0.35–0.52), 0.57 (95% CI: 0.46–0.70), and 0.34 (95% CI: 0.27–0.43), respectively (*P* < 0.001 for all).

**Table 3:** Hazard ratios (95% CI) for the association between time‐dependent exposure to antidiabetic medication classes during follow‐up and secondary endpoints

Secondary endpoint	Medication exposure	Events during unexposed, *n* (%)	Rate per 100py	Events during exposed, *n* (%)	Rate per 100py	Adjusted HR (95% CI)^a^	*P* value
HF hospitalization	Metformin	211	11.89	260	23.85	0.50 (0.41–0.60)	<0.001
	Sulfonylureas	234	16.56	237	16.33	0.61 (0.50–0.75)	<0.001
	Other	464	17.91	7	3.47	0.30 (0.14–0.64)	0.002
	Insulin	311	19.24	160	12.82	0.51 (0.40–0.64)	<0.001
CV‐related mortality	Metformin	178	11.24	106	4.93	0.50 (0.39–0.64)	<0.001
	Sulfonylureas	148	7.88	136	7.34	0.66 (0.50–0.85)	0.002
	Other	273	7.90	11	3.99	0.70 (0.33–1.47)	0.348
	Insulin	81	4.74	203	10.03	0.41 (0.30–0.57)	<0.001
All‐cause mortality	Metformin	184	8.43	346	22.33	0.43 (0.35–0.52)	<0.001
	Sulfonylureas	265	14.20	265	12.59	0.57 (0.46–0.70)	<0.001
	Other	520	14.99	10	3.82	0.38 (0.18–0.79)	0.009
	Insulin	140	8.23	390	19.22	0.34 (0.27–0.43)	<0.001

CI, confidence interval; CV, cardiovascular; HF, heart failure; HR, hazard ratio.

Adjusted analysis performed on multiply imputed data.

^a^ Adjusted for each medication listed in the table and HbA1c in a time‐dependent fashion, and the following baseline (time of index HF event) characteristics were age, sex, IMD category, BMI, smoking status, SBP, total cholesterol, HbA1c, history of CHD, history of ischaemic stroke, history of TIA, history of AAA, and history of PAD.

### Sensitivity analyses

In survivors after 30 days post‐HF diagnosis, the associations between the study outcomes and antidiabetic drug treatment persisted (*Table* *4*). However, the complete case analysis showed a weaker association with metformin (HR: 0.60; 95% CI: 0.44–0.82, *P* = 0.001) and a lack of association with sulfonylureas (HR: 0.85; 95% CI: 0.61–1.18; *P* = 0.329). The propensity score analysis showed consistent results, with HRs for metformin, sulfonylureas and insulin for the composite outcome of 0.46 (95% CI: 0.39–0.55), 0.67 (95% CI: 0.55–0.82), and 0.52 (95% CI: 0.43–0.65), respectively (*P* < 0.001 for all). Furthermore, controlling for temporal changes in diabetes control during follow‐up, as measured by the HbA1c level, did not alter the associations. Additional sensitivity analyses were consistent with the main analysis and are described in the supporting information.

**Table 4:** Sensitivity analyses, HRs (95% CI) for the association between time‐dependent exposure to antidiabetic medication classes during follow‐up, and the composite of cardiovascular death and HF hospitalization [primary endpoint]

Sensitivity analysis type	Exposure	Adjusted HR (95% CI)^a^	*P* value
Excluding first 30 days (32 subjects were censored within the first 30 days: 24 died, 8 lost) [*N* = 1140]	Metformin	0.49 (0.41–0.59)	<0.001
	Sulfonylureas	0.70 (0.58–0.84)	<0.001
	Other	0.49 (0.29–0.82)	0.007
	Insulin	0.55 (0.44–0.68)	<0.001
Complete case analysis [*N* = 393]	Metformin	0.60 (0.44–0.82)	0.001
	Sulfonylureas	0.85 (0.61–1.18)	0.329
	Other	0.21 (0.06–0.74)	0.015
	Insulin	0.59 (0.41–0.86)	0.007
Propensity score analysis^b^ [*N* = 1172]	Metformin	0.46 (0.39–0.55)	<0.001
	Sulfonylureas	0.67 (0.55–0.82)	<0.001
	Other	0.33 (0.17–0.66)	0.002
	Insulin	0.53 (0.43–0.65)	<0.001
Adjustment for time‐dependent HbA1c levels [*N* = 1172]	Metformin	0.50 (0.43–0.60)	<0.001
	Sulfonylureas	0.65 (0.54–0.78)	<0.001
	Other	0.41 (0.24–0.72)	0.002
	Insulin	0.54 (0.44–0.66)	<0.001
	HbA1c (per 10 mmol/mol increase)	1.04 (0.98–1.09)	0.217

A test against linearity for HbA1c provided no evidence (*P* = 0.460).

^a^ Adjusted for each medication listed in the table and HbA1c in a time‐dependent fashion, and the following baseline (time of index HF event) characteristics were age, sex, IMD category, BMI, smoking status, SBP, total cholesterol, HbA1c, history of CHD, history of ischaemic stroke, history of TIA, history of AAA, and history of PAD.

^b^ Analysis was performed using inverse probability of treatment weight (IPTW), adjusted for use of each of the other medication exposures listed in the table in a time‐dependent fashion. Variables used to create the propensity scores were age at index event, sex, ethnicity, BMI, fasted glucose, HbA1c, smoking status, total serum cholesterol, previous stroke, previous AAA, previous angina, and if ever prescribed insulin prior to index event.

## Discussion

Metformin is a guideline‐recommended therapy for the treatment of T2DM in HF.^1^ Despite evidence for a beneficial effect in this context,^2^ the evidence is more contentious in patients with T2DM and acute MI,^6^ and no studies have specifically investigated antidiabetic agent choice in patients with T2DM and HF of ischaemic aetiology. We interrogated the prospectively recorded EHRs of patients with T2DM and HF of ischaemic aetiology in England, including primary care data linked to hospital admission and mortality data. To our knowledge, this is the first study specifically investigating the association of antidiabetic therapy and outcomes in this cohort of patients.

We identified 1172 patients with T2DM and prior MI who had incident HF during the study period. Of the 50.9% who had a primary outcome event, most were taking metformin, which has previously been associated with reduced all‐cause mortality among patients with HF and T2DM.^18^ However, using adjusted analysis with drug class and HbA1c as time‐dependent variables, to account for temporal changes in medication use, periods of non‐use, and diabetes control, we observed a reduced hazard of both the composite and secondary endpoints for all major antidiabetic drug classes compared with not using the drug. These findings suggest that all included classes of antidiabetic agent, not just metformin, are associated with a reduced risk of cardiovascular mortality and HF hospitalization, which appears to be independent of temporal changes in diabetes control. However, the association was attenuated for sulfonylureas and insulin in complete case analysis, which may be a source of bias, and the results should therefore be interpreted with caution.

Potential reasons for this finding, which contrasts with previous literature suggesting better outcomes among metformin users,^2^, ^18^ might be two‐fold. Firstly, patients' medication usage may change over time, and therefore, events may be misclassified. Similarly, diabetes control varies over time. To account for this, we used antidiabetic medication and HbA1c levels as time‐dependent covariates to reflect periods of medication use and non‐use. Secondly, previous studies have included thiazolidinediones, which have known safety issues^12^, ^13^ and might therefore be a source of bias.

The European Society of Cardiology recommends metformin for possible safety and economic reasons, despite limited evidence.^19^ Our results show that metformin, sulfonylureas, and insulin are all associated with a reduction in the hazard of cardiovascular mortality and HF hospitalization, with no antidiabetic medication class having a clear association with greater benefit.

### Strengths and limitations

This study has several strengths. Firstly, the longitudinal study design and the analysis of antidiabetic medication and HbA1c as time‐varying covariates account for periods of use and non‐use, as well as diabetic control, during follow‐up. Secondly, using three linked electronic data sources, which are representative of the general population, maximizes the ascertainment of outcomes. Thirdly, medication status and baseline characteristics were recorded prospectively, prior to the development of HF, which limits the possibility of recall bias. Fourth, we accounted for the use of other antidiabetic medications in our analyses and removed patients taking thiazolidinediones, which risk introducing confounding due to their known harmful effects in HF. Finally, because we used a 90 day window to define discontinuation of medication, and most prescriptions are shorter than this, we account for ‘residual’ medication effects.

In this observational study, we can only report associations and not causal relationships as potential bias because of unmeasured confounding factors or indication bias might be present. Furthermore, although we adjusted for HbA1c level, we were not able to adjust for other parameters of diabetes control, which were unavailable. However, the baseline characteristics of patients with and without events were similar, both to each other and to other studies.^3^ Next, while we assumed that patients with prior MI and no history of HF had HF of ischaemic aetiology, we were unable to exclude other aetiologies. Finally, as with all such studies, we were unable to account for adherence or dosage information. Further strengths and limitations are addressed in the supporting information.

This study predates the use of newer treatments for T2DM such as sodium‐glucose‐co‐transporter‐2 inhibitors (several drugs of this class improve outcomes in HF, irrespective of the presence of T2DM^20^, ^21^, ^22^) and the GLP‐1 agonists and DPP‐4 inhibitors. Future research will focus on comparing these newer therapies with metformin to look for any potential differences in outcome.

## Conclusions

Acknowledging the limitations of observational studies and possible bias indicated by the complete case analysis, this study suggests that metformin, sulfonylureas, and insulin are associated with similar beneficial effects in patients with T2DM and HF of ischaemic aetiology. Defining the optimum regime of antidiabetic medication for patients with HF and T2DM is an outstanding question and should be the focus of future randomized studies.

## Conflict of interest

None declared.

## Funding

D.B. has received funding from a National Institute for Health Research Clinical Lectureship (CL‐2016‐17‐001) and the Academy of Medical Sciences (SGL020\1087). M.P.R. is employed by IQVIA, a research contract organization. H.H. is a National Institute for Health Research (NIHR Senior Investigator). This study is part of the BigData@Heart Consortium that is funded by the Innovative Medicines Initiative‐2 Joint Undertaking (grant agreement 116074). This Joint Undertaking receives support from the European Union's Horizon 2020 research and innovation programme and EFPIA; it is chaired, by DE Grobbee and SD Anker, partnering with 20 academic and industry partners and ESC. This work is supported by Health Data Research UK, which is funded by the UK Medical Research Council, Engineering and Physical Sciences Research Council, Economic and Social Research Council, Department of Health and Social Care (England), Chief Scientist Office of the Scottish Government Health and Social Care Directorates, Health and Social Care Research and Development Division (Welsh Government), Public Health Agency (Northern Ireland), British Heart Foundation, and Wellcome. This study was supported by National Institute for Health Research (RP‐PG‐0407‐10314) and Wellcome Trust (086091/Z/08/Z). This study was supported by the Farr Institute of Health Informatics Research at UCL Partners, from the Medical Research Council, Arthritis Research UK, British Heart Foundation, Cancer Research UK, Chief Scientist Office, Economic and Social Research Council, Engineering and Physical Sciences Research Council, National Institute for Health Research, National Institute for Social Care and Health Research, and Wellcome Trust (MR/K006584/1). This paper represents independent research part funded (A.G.I. and S.D.) by the National Institute for Health Research (NIHR) Biomedical Research Centre at University College London Hospitals.

## Supporting information




**Table S1.** STROBE and RECORD checklists.
**Table S2.** Sensitivity analyses for antidiabetic drugs post index HF event and prior to censorship, and HR (95% CI) of exposure vs. no exposure for primary outcome.
